# Mechanical properties and failure behavior of unidirectional porous ceramics

**DOI:** 10.1038/srep24326

**Published:** 2016-04-14

**Authors:** Jordi Seuba, Sylvain Deville, Christian Guizard, Adam J. Stevenson

**Affiliations:** 1 Laboratoire de Synthèse et Fonctionnalisation des Céramiques, UMR3080 CNRS/Saint-Gobain, F-84306 Cavaillon, France; 2 Institut Européen des Membranes, Université de Montpellier 2, Place Eugéne Bataillon, 34095 Montpellier Cedex 5, France

## Abstract

We show that the honeycomb out-of-plane model derived by Gibson and Ashby can be applied to describe the compressive behavior of unidirectional porous materials. Ice-templating allowed us to process samples with accurate control over pore volume, size, and morphology. These samples allowed us to evaluate the effect of this microstructural variations on the compressive strength in a porosity range of 45–80%. The maximum strength of 286 MPa was achieved in the least porous ice-templated sample (P(%) = 49.9), with the smallest pore size (3 *μ*m). We found that the out-of-plane model only holds when buckling is the dominant failure mode, as should be expected. Furthermore, we controlled total pore volume by adjusting solids loading and sintering temperature. This strategy allows us to independently control macroporosity and densification of walls, and the compressive strength of ice-templated materials is exclusively dependent on total pore volume.

Macroporous ceramics are widely used in applications such as filtration, thermal insulation, scaffolds for tissue engineering, oxygen transport membranes, and solid oxide fuel cells^1^,^2^. They must combine mechanical stability with at least one other functional property such as high permeability, low thermal conductivity, or biocompatibility.

Strength is also an important parameter. It is usually increased by decreasing the total pore volume, even though it may degrade other functional properties. Morphological parameters such as pore size, morphology, or tortuosity can become crucial to maximize the performance while maintaining high strength. For example, a significant improvement in strength can be achieved by engineering anisotropic structures to mechanically reinforce the direction of the principle stress. However, most of the techniques conventionally used to produce macroporous ceramics with aligned porosity do not offer this level of flexibility.

Ice-templating is a processing technique suitable for producing anisotropic macroporous materials. It is based on freezing of colloidal suspensions and segregation of particles by the solidification front. After solidification, the frozen solvent is removed, leaving pores whose morphologies are a replica of the sublimated crystals^3^. Finally, the green body is sintered to consolidate the microstructure. This process provides control of the pore architecture (pore volume, size, and morphology) through initial solids loading, cooling rate, or additives. Therefore, a good understanding of these parameters is essential to understanding the relationships between processing, microstructure, and the mechanical properties of anisotropically porous materials to extend their use to the aforementioned applications.

As predicted by Gibson and Ashby^4^,^5^,^6^, pore architecture greatly determines the mechanical response of porous materials. Identifying the failure mechanisms that govern fracture can lead to more optimized architectures and better performance. However, ice-templated materials have more complex structures than the models proposed in references^4^,^5^,^6^. Recently, different groups studied the mechanical properties of ice-templated materials based on their pore structure. For example, Ojuva *et al*.^7^ investigated the effect of solids loading and freezing temperature on zeolite materials and related the compressive strength to the pore aspect ratio. Porter *et al*.^8^ found a similar result in *TiO*_2_ by controlling the pore size through the polyethylene glycol (PEG) viscosity, pH, and isopropanol alcohol (IPA) concentration. Hunger *et al*.^9^ described the mechanical behavior of a chitosan-hydroxiapatite composite using an hybrid model accounting for the unidirectional pores and the porosity in the walls. However, these studies are mostly limited to a highly porous materials (>80%) and a more detailed work describing the microstructural effects on compressive strength of unidirectional porous ceramics in a broader porosity range is still lacking.

Our objective is to characterize the compressive strength of ice-templated ceramics in a broad porosity range and link their mechanical behavior to the pore architecture. We will discuss the effects of pore volume, size, and morphology on the failure mechanism. This discussion will allow us to assess the validity of the Gibson and Ashby models for the prediction of the compressive strength of unidirectional porous materials.

## Experimental Procedure

### Sample preparation

Ice-templated specimens were produced following the same procedure reported elsewhere^10^. Basically, suspensions were prepared by mixing distilled water with 3 mol% yttria-stabilized zirconia (TZ-3YS, Tosoh, Tokyo, Japan) at different weight ratios (from 45% to 70%), 0.75 wt.% of dispersant (Prox B03, Synthron, Levallois-Paris, France), and 3 wt.% of organic binder PVA (PVA2810, Wacker, Burghausen, Germany). In some suspensions, zirconium acetate (20 g/L) was added to the slurry to modify the pore morphology. Afterwards, the slurry was magnetically stirred to ensure a good dispersion and ball milled for a minimum of 18 h to break up the agglomerates. It was then deaired for at least 10 min.

The ice templating process consisted of pouring 10 ml of slurry into a PTFE mould (20 mm diameter and 25 mm height) placed on a copper plate and freezing from the bottom to the top. The top of the samples was exposed to air and kept at room temperature. The freezing temperature was controlled by circulating silicone oil regulated by a cryothermostat (Model CC 905, Hubert, Offenburg, Germany). The cooling rate was set at 2 °C/min. A faster cooling rate was achieved dipping a copper rod with the mould on top in liquid nitrogen. The cooling rate was monitored with a thermocouple and it was determined to be 25 °C/min on average. After solidification, samples were demoulded and sublimated for at least 48 h in a commercial freeze-dryer (Free Zone 2.5 Plus, Labconco, Kansas City, Missouri, USA).

Binder was removed from the green bodies by heating to 500 °C at 3 °C/min with a 5 h hold. Then, samples were sintered between 1300 °C and 1400 °C at 5 °C/min and held 3 h. The cooling rate was kept constant at 5 °C/min until room temperature.

Samples with a non-oriented porosity were also prepared. The same zirconia powder was mixed with a commercially available pore former polypropylene (Propyltex 140S, Micro Powders Inc, Tarrytown, USA) at different ratios (50 wt.%, 60 wt.%, and 70 wt.%) and mixed with distilled water. The slurry was magnetically stirred and ball milled for a minimum of 24 h to break up the agglomerates. Afterwards, the slurry was frozen by dipping the container in liquid nitrogen and freeze-dried to obtain an homogeneous mix. Then, 8 g of the powder was pressed at 0.8 MPa in a mould of 20 mm diameter. The sintering temperature and dwell time were the same as those used for the ice-templated samples. To ensure a proper burn-out and avoid the formation of cracks an extra hold was added at 900 °C.

### Morphological characterization

The overall porosity, P(%), was calculated based on the mass (*m*) and volume (*V*) of the samples with respect to that of fully dense TZ-3YS (*ρ*_*ysz*_ = 5.8 *g*/*cm*^3^), as:


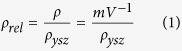



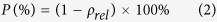


The results were confirmed in at least two samples per condition by the Archimedes method (ASTM B962-13). The deviation between the overall porosity obtained by Archimedes and by Eq. 2 ranged 1–2%. The determination of pore size and wall thickness distributions were performed by image analysis using the “Local thickness” plug-in of the Fiji software^11^. In order to obtain a representative value of pore size and wall thickness, a minimum of 5 images per sample were analyzed. All the images were taken at different locations in a cross section perpendicular to the freezing direction (7 mm from the bottom of the sample) with a scanning electron microscope (Nova NanoSEM 230, FEI, Hillsboro, USA)) at 10–15 kV. The results were confirmed by mercury intrusion porosimetry (AutoPore IV 9500, Micromeritics), 2 samples per condition, with an applied pressure up to 0.31 bar.

### Mechanical characterization

The mechanical properties of specimens with isotropic (pore formers) and anisotropic (ice-templated) porosity were measured by a compression test (LR15K Plus, Lloyd Instruments, Meerbusch, Germany) at a crosshead speed of 0.5 mm/min. The bottom and the top of the samples were removed with a slow speed saw leaving the final dimensions around 15 mm diameter and 17 mm height. Samples were tested with a cardboard pad on both sides to minimize the effect of superficial defects and misalignment. In all the tests, the maximum load at the end of the elastic stage was used to calculate the compressive strength.

## Results and Discussion

**Figure 1:**
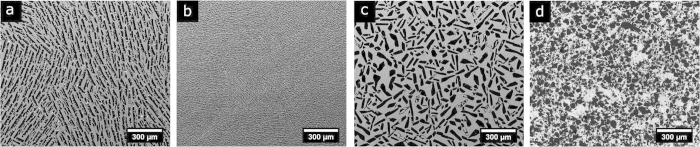
SEM cross-sections of ice-templated samples (**a**) frozen at 2 °C/min, (**b**) frozen at 25 °C/min, (**c**) frozen at 2 °C/min with zirconium acetate (ZRA), and (**d**) made by pore formers. In all cases the total pore volume was around 51%.

We investigated the effects of pore volume, size, morphology, and directionality on the compressive behavior of porous materials. Figure 1 shows the representative microstructures of the studied samples.

### Microstructural control

The typical lamellar microstructure obtained in ice-templated samples when water is used as a solvent it is shown in Fig. 1a. Total pore volume P(%) control was achieved by adjustment of the solids loading, as reported in Table 1. Increasing the solids loading from 50% to 65% caused a decrease in P(%) from 69.5% to 52.5%. This effect is independent of the freezing rate used. Thus, samples ice-templated at different freezing rates but with the same initial solids loading have a comparable total pore volume. For example, specimens in Fig. 1a,b exhibited a pore volume of 52.5% and 51.5% although they were frozen at different rates, 2 °C/min and 25 °C/min respectively. These results are consistent previous results for the same materials and similar processing conditions^12^,^13^,^14^,^15^,^16^.

**Figure 2:**
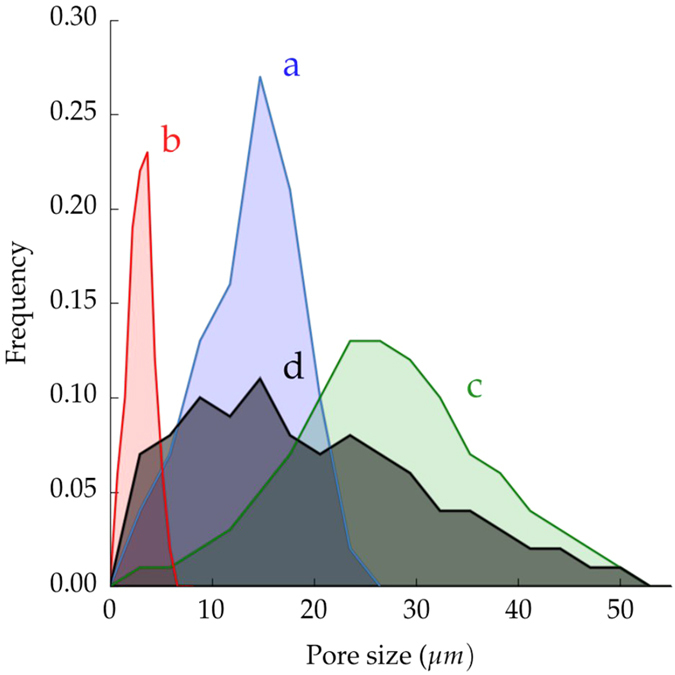
Pore size distribution obtained by image analysis of samples shown in Fig. 1. Ice-templated samples (**a**) frozen at 2 °C/min, (**b**) frozen at 25 °C/min, (**c**) frozen at 2 °C/min with zirconium acetate (ZRA), and (**d**) made by pore formers.

For ice-templated materials, the freezing rate is the most important feature controlling pore size. Figure 2 shows the pore size distributions of the samples described in Fig. 1 and Table 1. Sample frozen at 25 °C/min (Fig. 1b) exhibits a smaller mean pore size (3.1 ± 1.2 *μ*m) and a narrower distribution compared with the sample frozen at 2 °C/min (13.7 ± 4.8 *μ*m), Fig. 1a. The magnitude of supercooling ahead of the ice front increases with freezing rate, while the tip radius of the ice crystals decreases, thus creating a finer microstructure^17^.

We measured pore size distribution using two different techniques: image analysis (*d*_*pIA*_) and mercury porosimetry (*d*_*pHg*_), Table 1. Both gave comparable results in all the experimental conditions with the exception of the specimen frozen at 2 °C/min and 65 wt.% solids loading. Most likely, increasing the particle concentration in the solution increases the probability of creating closed pores or bottlenecks that affect the measurement by mercury porosimetry but does not necessarily affect the image analysis.

The freezing rate is not the only parameter affecting the mean pore size. The comparison of samples with different solids loading and frozen at constant cooling rate, either 2 °C/min or 25 °C/min, shows a decrease in pore size when the solids loading increases, Table 1. However, this pore size reduction is of secondary importance compared to the effects of the cooling rate. The main influence of solids loading on pore size variation is increasing the number of particles rejected by the solidification front. When the solids loading increases, it becomes more difficult for the advancing solidification front to repel the particles, thus hindering the ice growth and eventually reducing the final pore size.

Pore morphology is primarily defined by the nature of the solvent^18^. Many solvents have been used before: water^17^,^19^, camphene^12^,^13^,^20^,^21^, TBA^22^,^23^,^24^, cyclohexane^25^. In the case of water, pores usually exhibit a lamellar morphology, (Fig. 1a). However, the addition of components such as glycerol^26^,^27^, polystyrene^20^, or sucrose^26^ can modify the pore size and shape.

Recently, it has been reported that the addition of zirconium acetate (ZRA) into the initial slurry turns the pore morphology into a honeycomb-like structure with smooth surfaces^28^. ZRA modifies the ice-crystal morphology by affecting incorporation of water molecules into the growing ice crystal. Even though the pore morphology changes (Fig. 1c), the fundamental mechanism for pore formation remains unaltered, and therefore, it is possible to control the pore morphology independently of the total pore volume. However, the addition of ZRA modifies the pore size distribution, even if the solids loading and the freezing rate used are identical to samples processed without ZRA, Fig. 2.

A set of samples with isotropic porosity (Fig. 1d) was also prepared by organic burn-out (pore formers). Since the macroporosity obtained by this technique is a replica of the organic part originally mixed with the ceramic powder, the total pore volume is controlled by the organic-ceramic ratio. Pores are homogeneously dispersed with no preferential orientation, leaving an isotropic material. The processing parameters (volume and size of organic, and sintering temperature) were adjusted to obtained a total pore volume of 49.6% and a mean pore size 19.8 ± 11.9 *μ*m, similar to the samples obtained by ice-templating. However, Fig. 2 shows that the pore size distribution is remarkably broader than the ice-templated samples.

### Prediction by mechanical models

**Figure 3:**
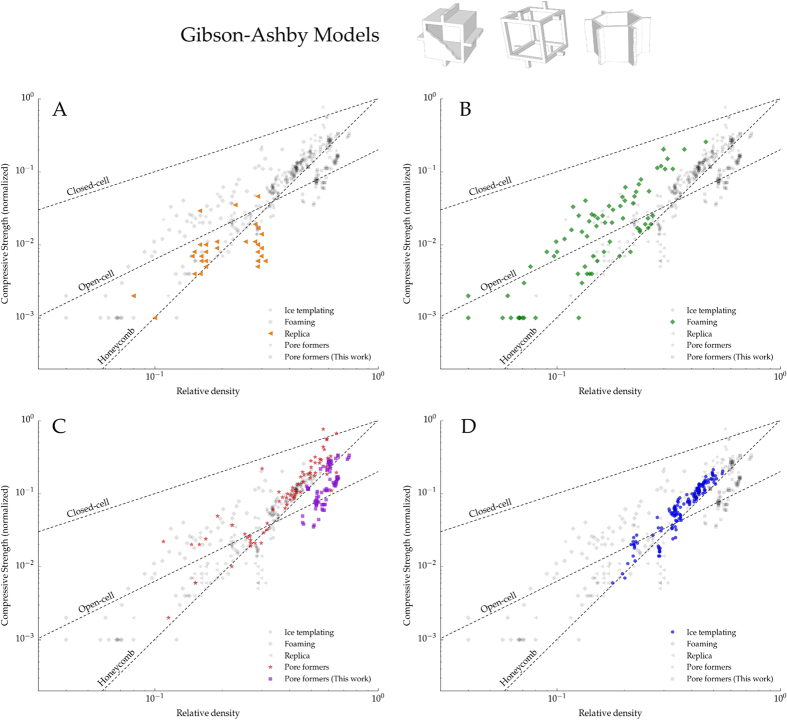
Each plot in the panel highlights the compressive strength data versus relative density for four different macroporous ceramics processed by: (**A**) Replica, (**B**) Foaming, (**C**) Pore formers, and (**D**) Ice-templating. The experimental points of the other techniques are also shown in grey for comparison. The dotted lines represent the models proposed by Gibson and Ashby: Closed-cell (Eq. 3), Open-cell (Eq. 4), and Honeycomb out-of-plane (Eq. 5). Blue points in (**D**) and purple in (**C**) correspond to experimental points obtained in this work and the rest have been extracted from^29^.

The most widely used models to predict the mechanical response of cellular materials were developed by Gibson and Ashby^4^,^6^. These models differentiate between three morphologies: open-cell, closed-cell, and honeycomb (Fig. 3). Although these models have been applied to different mechanical properties such as fracture toughness, creep, or Young modulus, we discuss only the compressive strength described by:

• Closed-cell (brittle crushing)


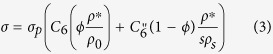


where *C*_6_ = 0.65 and 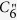 = 1 are geometric constants determined empirically, *ϕ* represents the solid fraction in the edges, *ρ*_*s*_ and ρ^*^ are the apparent density of the dense and cellular material respectively, and *σ*_*p*_ the modulus of rupture of the solid material within the walls.

• Open-cell (brittle crushing)


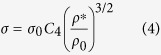


with *C*_4_ = 0.2, and where *σ*_*p*_, *ρ*_*s*_, and ρ^*^ have the same meaning as in the closed-cell model.

• Honeycomb (out-of-plane)


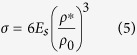


where *E*_*s*_ is the Young’s modulus of the corresponding dense material, and *ρ*_*s*_ and ρ^*^ are again the apparent density of the dense and cellular material respectively.

Figure 3 shows the normalized compressive strength of samples processed in this work along with those obtained by other processing methods (replica, foaming, and pore formers) extracted from^29^. In all cases, the strength was normalized by the flexural strength of the bulk material because bending has been identified as the main failure mechanism in cellular materials within the porosity range studied here^30^. This normalization allows us to isolate the effect of pore microstructure as we assess the mechanical models.

Figure 3A shows that the strength of samples obtained by the replica method always have a lower strength value than that predicted by Eq. 4. This behavior has been extensively reported in the literature^31^,^32^,^33^,^34^ and it is caused by the presence of microstructural flaws in the struts created during the burn-out of the organic template.

On the other hand, Fig. 3B,C show that the strength of specimens processed by either foaming or pore formers fell in the broad range predicted by the closed (Eq. 3) and open cell models (Eq. 4). The macroporosity obtained at low relative density by both techniques better resemble the open-cell structure, and thus Eq. 4 becomes the best descriptor of the compressive strength. Nevertheless, it is important to highlight the high variability of the data in this range, probably due to microstructural defects such as: cracks originated during burn-out or drying, presence of closed pores, inclusions, or dispersion in pore size distribution. When the relative density increases, the proportion of closed pores increases accordingly and the specimens exhibited a compressive strength near the upper limit determined by Eq. 3. Interestingly, the specimens obtained by pore formers fell in the prediction made by the honeycomb-out-of-plane model (Eq. 5). This effect can be related with the variation in load distribution through the structure when the porosity decreases. When the number of closed pores increases the structure tends to fail more by buckling, as predicted by honeycomb model^6^, instead of bending of the struts, like in the open-cell model^4^.

**Figure 4:**
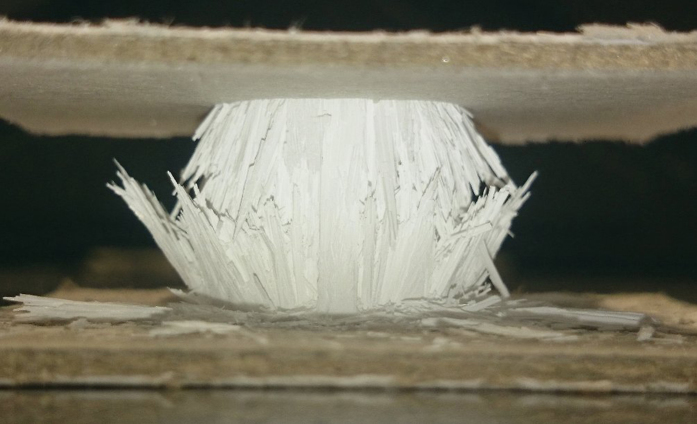
Detail of a buckling fracture in an ice-templated sample.

Figure 3D shows that ice-templated architectures are better described by the honeycomb out-of-plane model (Eq. 5). Figure 4 shows a broken sample that failed perpendicular to the external load at the middle of the specimen, where the stresses reach the maximum value, and indicative of a buckling fracture. The combination of anisotropic porosity with perpendicular struts connecting the main walls, characteristic of the ice-templated materials, interlock their lateral motion and prevent the shear stresses. Similarly as it was previously explained for porous materials obtained by pore formers, the ice-templated structure becomes more prone to collapse by buckling of the walls instead of localized bending as it was identified in foams^4^,^32^,^35^. Buckling also has been reported to be the main failure mechanism in different ice-templated materials^7^,^9^ and in other unidirectional porous materials processed by wood pyrolisis^36^, extrusion^37^, or 3D printing^38^.

**Figure 5:**
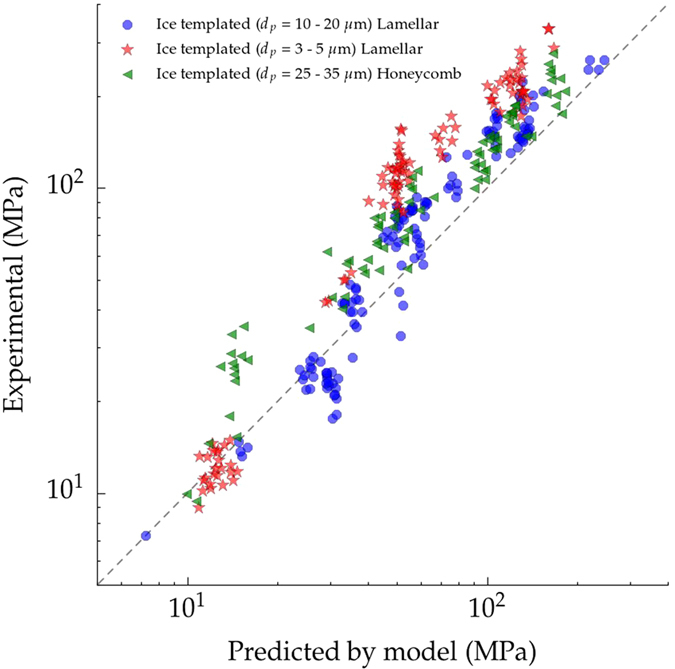
Parity plot comparing the experimental and the theoretical strength predicted by Eq. 5. The three sets of samples have been ice-templated at: 2 °C/min with lamellar morphology (blue), 2 °C/min with honeycomb morphology (green), and 25 °C/min with lamellar morphology (red).

Figure 5 shows the compressive strength obtained experimentally versus the strength predicted by Eq. 5 for three sets of ice templated samples: lamellar frozen at 2 °C/min (Fig. 1a), lamellar frozen at 25 °C/min (Fig. 1b), and honeycomb frozen at 2 °C/min (Fig. 1c). The model describes the compressive behavior remarkably well for both pore morphologies. Although the addition of zirconium acetate modifies the pore morphology towards a honeycomb structure, the ceramic walls are still continuous and oriented parallel to the direction of the load, and therefore the predominant failure mechanism is still buckling. As we showed previously^39^, when the continuity of the walls decreases, like in the dendritic porosity created when camphene is used as a solvent, Eq. 5 is not a good descriptor of the strength. The validity of the model is thus mainly determined by the directionality and continuity of the walls rather than their cross sections. Nevertheless, the Gibson and Ashby models were initially derived from the response of an idealized unit cell to a stress and do not consider microstructural effects such as particle size, microstructure of the struts, interaction between pores, or pore size. The effects of microstructure can be observed in Fig. 5 where Eq. 5 underestimates the strength of ice-templated samples with smaller pores (3–5 *μ*m). This effect is particularly remarkable at high relative density (i.e. high strength) where the amount of energy stored during compression is higher and materials tend to be more brittle. In contrast, at low values of relative density, the total pore volume and the presence of microstructural defects increase and favor the progressive damage by buckling of the struts and the fit by Eq. 5 becomes more accurate.

### Compression tests

Compressive strength is the most reported mechanical property for ice-templated materials due to its simplicity in testing and sample preparation. Nonetheless, it is difficult to compare with the results in the literature because structures are notably different in terms of size, morphology, directionality, and even microstructure of the walls. However, it is possible to point out several trends common in the majority of samples.

#### Effect of solids loading, pore size, and pore morphology

**Figure 6:**
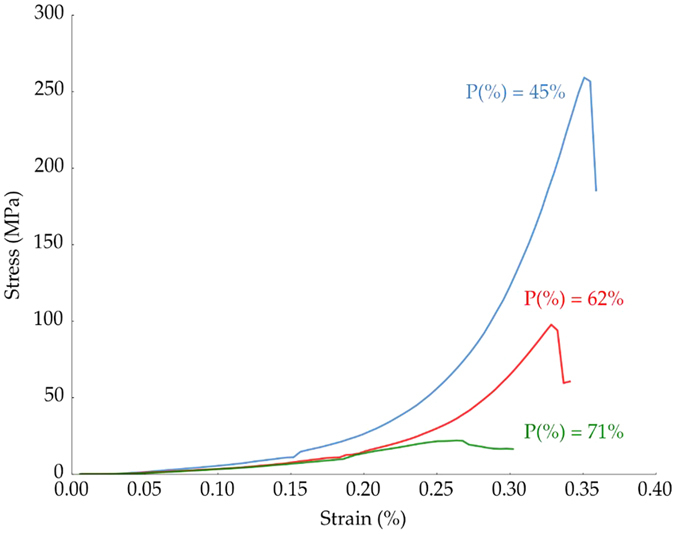
Effect of total pore volume on the compressive behavior.

Representative stress-strain curves recorded during the compression test are shown in Fig. 6 for three ice templated samples with different pore volumes, average pore size of 10–20 *μ*m, and lamellar morphology. All specimens were tested with the pores aligned in the direction of the applied load. The P(%) = 45% and 62% samples exhibited an initial linear stage up to a sudden rupture, typical of brittle behavior. In contrast, samples with higher porosity (P(%) = 71%) presented a cellular-like rupture commonly observed in ceramic foams: after an initial elastic phase, the stress reaches a steady value caused by the progressive collapse of the struts^40^.

**Figure 7:**
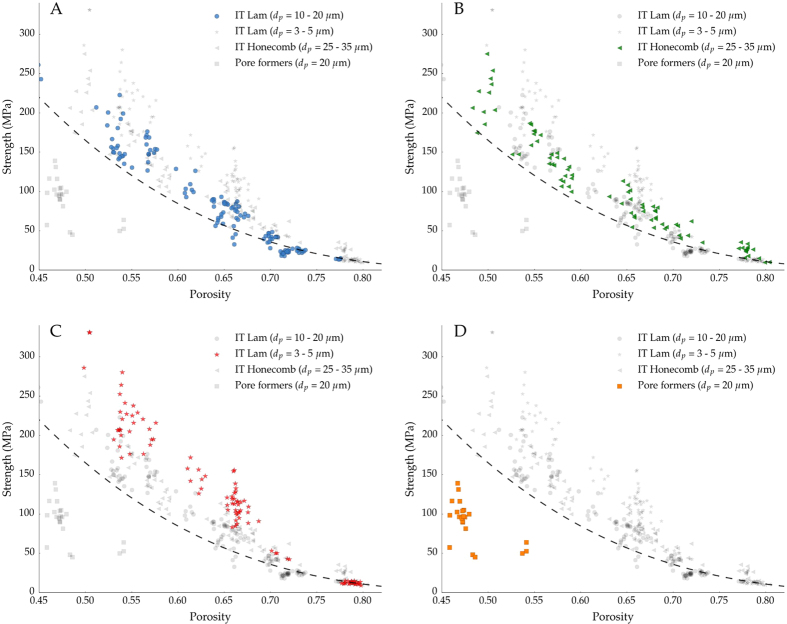
Compressive strength dependence on porosity in ice templated samples frozen at 2 °C/min (**A**), 25 °C/min (**C**), and with a honeycomb morphology (**B**). Isotropic samples made in this work by sacrificial method are also represented (**D**). The dashed line corresponds to the Honeycomb out-of-plane model shown in Eq. 5. The experimental points of the other conditions are represented in grey for comparison.

Figure 7 shows the effect of total pore volume (from 45% to 80%), size (Fig. 7A vs. Fig. 7C), morphology (Fig. 7A vs. Fig. 7B), and directionality (Fig. 7A vs. Fig. 7D) on compressive strength. As expected, when the total pore volume decreases the compressive strength increases independent of the pore microstructure and architecture.

Although samples with isotropic porosity (pore formers) processed in this work have lower strength than the ice-templated ones (Fig. 7A,D), we must be careful to evaluate the particular role of pore directionality. As Fig. 3 shows, the structures that obey the honeycomb out-of-plane model tend to collapse at higher strength than those that fail by localized bending or shear (open-cell) at relatively high relative density. The parallel alignment of the walls with the load allows a more optimal distribution of the stresses and prevents the bending of the struts at lower loads. Therefore, we can hypothesize than the optimized microstructure of ice-templated materials explains this strength increment.

However, there are other microstructural factors that also affect the mechanical performance of the isotropic samples such as the presence of large agglomerates (Fig. 1d) or the broad pore distribution (Fig. 2). Both features certainly might impact the strength through the existence of weaker points and stress concentrators.

Samples with lamellar and honeycomb structure (Fig. 7A,B) followed a similar trend, and therefore compressive strength seems to be unaffected by the pore morphology. In contrast, samples with smaller pore size (Fig. 7C) have a higher strength than their counterparts with larger pores and similar pore volume. This effect becomes less important when the pore volume increases, even reaching a point (around 75%) where the strength seems unaffected by the pore size. Although the influence of pore size has been extensively reported in different types of porous ceramics^8^,^15^,^41^,^42^,^43^, the main cause of this behavior is unclear and it might result from a combination of factors. One of the main contributions could be related with reduction of the volume of the struts. For a constant pore volume, a decrease in pore size will leads to a wall thickness reduction, affecting the probability of finding a catastrophic defect and the strength^10^,^44^. The strength of ceramics is strongly related with the volume of the material solicited, it is therefore more convenient to describe their mechanical properties based on the distribution of the solid parts (struts) rather than the organization of the voids (pores). For this reason it is more accurate to refer this strengthening effect as a wall thickness effect instead of pore size.

Interestingly, when the pore volume increases the experimental strength values tend to those predicted by the honeycomb out-of-plane model, regardless of the pore structure. This behavior might be related with the transition between failure mechanisms (brittle and cellular-like fracture)^30^. When the pore volume increases above a certain value (typically above 60%), samples tend to fail progressively after the local buckling of the struts and pore morphology and size become secondary parameters to optimize to increase the strength. When the pore volume decreases, the material fails in a brittle manner with cracks propagating parallel to the load, and microstructural parameters like wall thickness distribution are of primary importance to increase the strength with no further reduction on the pore volume.

#### Effect of sintering temperature

**Figure 8:**
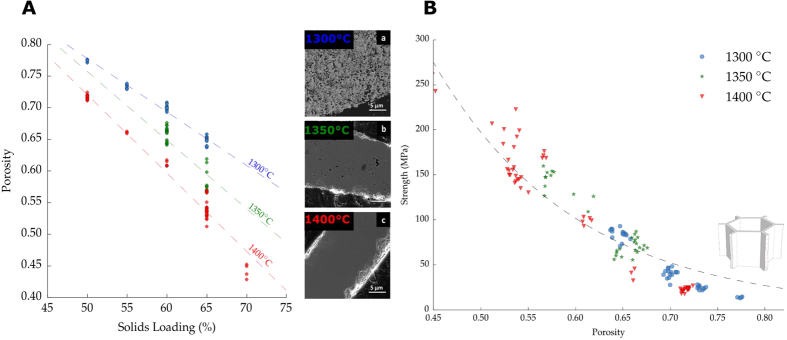
(**A**) Effect of solid loading and sintering temperature on % porosity and evolution of wall densification. (**B**) Relation between compressive strength and total pore volume for ice-templated samples frozen at 2 °C/min, with a pore size in the range of 10–20 *μ*m, and sintered at different temperatures (1300–1400 °C). Dashed line represents the honeycomb out-of-plane model, Eq. 5.

Total pore volume can also be tailored by the sintering temperature. Figure 8A shows the effect of solids loading and sintering temperature on total pore volume P(%). The solids loading investigated ranged between 50 wt.% and 65 wt.% for three different sintering temperatures (1300 °C, 1350 °C, and 1400 °C). Although, both parameters can modify P(%), the nature of the variation is different in each case. The spaces between the walls (interlamellar porosity) are determined for the most part by the ice templating process and it is a direct consequence of the solids loading and the sublimation of the ice crystals. Alternatively, porosity in the ceramic walls (intralamellar porosity) is mainly determined by the densification within the walls and therefore controlled by the sintering temperature. The SEM micrographs in Fig. 8A show the microstructural evolution in the walls when the sintering temperature increases from 1300 °C to 1400 °C for a given solids loading (65 wt.%). At 1300 °C (Fig. 8A) the residual porosity was estimated by image analysis to be around 24%, significantly higher compared to the 12% of the samples sintered at 1400 °C (Fig. 8C).

**Figure 9:**
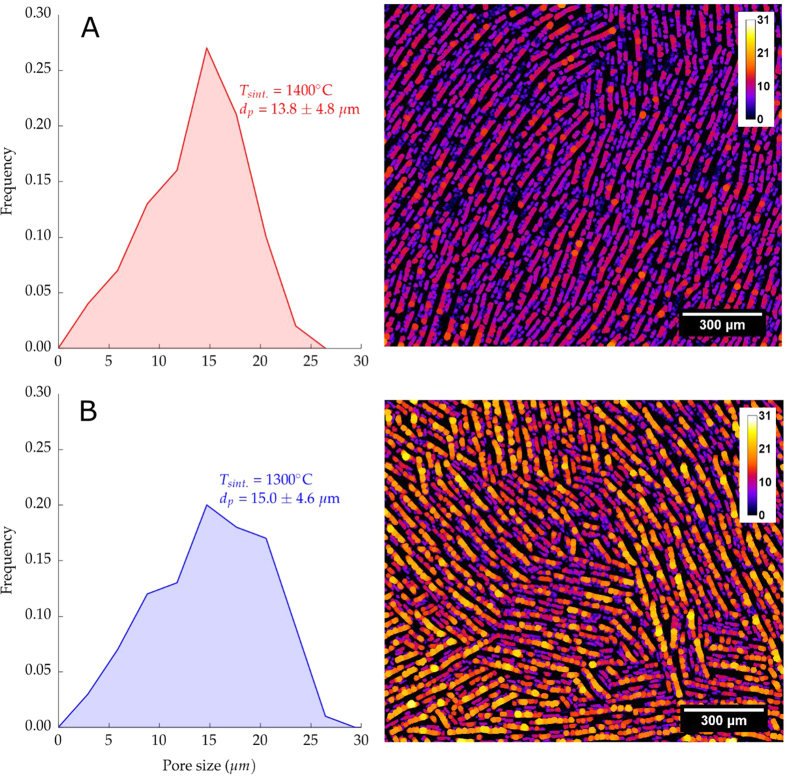
Image analysis showing the pore size population of ice templated samples frozen at 2 °C/min, 65 wt.% solids loading and sintering temperature (**A**) 1400 °C (**B**) 1300 °C.

The higher densification of the walls at 1400 °C increases the observed shrinkage, Fig. 9. Samples sintered at 1300 °C exhibited a radial shrinkage of 17%, significantly lower than the 21% obtained at 1400 °C. At higher sintering temperatures, the densification of the walls drives the shrinkage of macropores and therefore the pore size distribution becomes narrower. This behavior can be observed in the image analysis performed at samples sintered at 1400 °C and 1300 °C and shown in Fig. 9.

Interestingly, the relative reduction of pore volume caused by the sintering temperature is remarkably different depending on the initial wall thickness, and hence the particle concentration used. For example, samples in Fig. 8A with 65 wt.% exhibit a porosity reduction around 20% when the sintering temperature increases from 1300 °C to 1400 °C. In contrast, at 50 wt.% solids loading the reduction of the total porosity is around 8% for the same variation in temperature.

However, no additional increment in the strength has been observed caused by the reduction on the residual porosity in the walls at a given total pore volume, (Fig. 8B). All the samples, independently of the sintering temperature, followed the same relationship between strength and porosity as predicted by Eq. 5. This suggests that the compressive strength of ice-templated materials is determined mainly by the total pore volume, rather than the strength of the individual struts. This result is contrary with Li *et al*.^45^, that reports an exclusive effect of interlamellar porosity on the strength of ice-templated materials. Nevertheless, further work should be made in that regard to assess the particular role of inter- and intralamellar porosity on mechanical properties.

## Conclusions

This work reveals that the compressive behavior of unidirectional porous ceramics can be predicted by the honeycomb out-of-plane model in a wide porosity range, from 45% to 80%. However, the use of the model is limited to structures with a continuous array of unidirectional walls and where buckling is the main failure mode. Further improvements of the model considering microstructural effects on the strength like the pore size reduction could lead to more accurate results.

In addition, ice-templating exhibits a high versatility to control almost independently the main pore descriptors (volume, size, and morphology). The possibility to aim for a specific porosity, and hence strength, (through the solids loading) and tailor the densification of the walls (through the sintering temperature) is a powerful tool to adjust the specific surface area of the walls while keeping a constant pore volume. This flexibility can be useful in applications like SOFC cathodes or tissue engineering scaffolds, where a sufficient amount of macroporosity (interlamellar porosity) is required to guide the flow of the fluid, coupled with intralamellar porosity to improve conversion efficiency^46^ or cell proliferation^47^.

## Additional Information

**How to cite this article**: Seuba, J. *et al*. Mechanical properties and failure behavior of unidirectional porous ceramics. *Sci. Rep*. **6**, 24326; doi: 10.1038/srep24326 (2016).

## Figures and Tables

**Table 1:** Summary of the most relevant pore descriptors and their respective ice-templating conditions.

Solidloading(wt.%)	Freezing rate(°C/min)	Porosity(%)	Mean *d*_*pHg*_(*μ*m)	Mean *d*_*PIA*_(*μ*m)	Mean wallthickness(*μ*m)
50%	2	69.5%	19.1	20.0 ± 8.5	11.2 ± 4.5
55%	2	64.2%	17.5	17.7 ± 6.7	14.7 ± 6.2
60%	2	60.4%	14.2	13.9 ± 5.5	15.8 ± 6.8
65%	2	52.5%	8.3	13.7 ± 4.8	19.1 ± 8.2
50%	25	70.4%	5.4	4.7 ± 1.9	3.6 ± 1.5
55%	25	65.2%	4.3	4.3 ± 1.7	3.9 ± 1.6
60%	25	60.7%	3.9	4.1 ± 1.7	5.2 ± 2.2
65%	25	51.5%	2.9	3.1 ± 1.2	3.0 ± 1.3

*d*_*pHg*_ and *d*_*pIA*_ correspond to the mean pore size obtained by mercury porosimetry and image analysis respectively.
